# Mycobacterium culturing and drug resistance of osteoarticular tuberculosis in Xinjiang, China

**DOI:** 10.1097/MD.0000000000019697

**Published:** 2020-04-17

**Authors:** Yeerzati Hajiaheman, Yi Yang, Nuerhanati Shayilanbieke, Gele Jin

**Affiliations:** Department of Orthopaedics, The First Affiliated Hospital of Xinjiang Medical University, Urumqi, Xinjiang, China.

**Keywords:** culturing, drug resistance, mycobacterium, osteoarticular tuberculosis

## Abstract

This study aims to elucidate the strains and drug resistance of mycobacterium isolated from osteoarticular tuberculosis (OATB) patients and provide a reference for the diagnosis and treatment of OATB.

Sixty-nine clinically diagnosed and surgically treated OATB patients were collected in time period of January 2017 to December 2018 at the First Affiliated Hospital of Xinjiang Medical University. The BACTEC MGIT 960 system was used for mycobacteria culturing, strain identification, and drug susceptibility testing, and the mycobacteria culture positive rate, species distribution, and drug resistance were analyzed.

Within 4 weeks, 24 (34.78%) isolates of mycobacteria culture were positive; 40 (57.97%) isolates were positive, when culturing time was expanded to 8 weeks, and the difference was statistically significant (*P* < .05). Among the 40 isolates, 24 (60%) were identified as mycobacterium tuberculosis (MTB), 10 (25%) were *Mycobacterium bovis*, and 6 (15%) were non-tuberculous mycobacteria (NTM). Among total 69 isolates, 40 were enrolled in drug sensitivity test, and 15 (37.5%) isolates were confirmed drug resistant strains, in which 5 isolates were MTB, 4 isolates were *M. bovis*, and 6 isolates of NTM.

The pathogen of clinically diagnosed OATB was mainly MTB. However, *M. bovis* and NTM also accounted for a considerable proportion, and their drug resistance rate was higher. Extending the culturing time appropriately could improve the culture positive rate. NTM was a drug resistant strain, and mycobacteria culturing, strain identification, and drug resistance analysis should be carried out to serve as a guide for individual treatment.

## 1 Introduction

Tuberculosis (TB) is one of the major infectious diseases that causes 1.5 million people's deaths each year worldwide. China ranks as the third highest TB burden country in the world with about 1 million incident cases each year.^[1]^ In Xinjiang, due to the special climate, geographical environment, and population characteristics, the incidence of TB is significantly higher than the national average level.^[2]^

Osteoarticular tuberculosis (OATB) is the third most common infection of extra-pulmonary TB diseases after pleural and lymphatic TB which is associated with a high morbidity and mortality rate.^[3]^ However, as OATB is a paucibacillary condition, no systematic study on the bacterial characterization, especially culturing, identification, and drug resistance analysis of pathogens, has been completed.

Culturing of mycobacteria for strain identification and anti-TB drug susceptibility testing plays a critical role in the diagnosis and treatment of TB.^[4]^ Liquid medium is user-friendly and time-saving, and its culture-positive rate is higher. However, it is currently used only for research.^[5]^ Given the aforementioned situation, we used BACTEC MGIT 960 system in this study.

Our research team funded by national science and technology project conducted a leading, in-depth research on the bone and joint TB in Xinjiang. In our hospital, there were about 1000 cases of OATB patients treated surgically in the past 5 years. According to a report by the Chinese Center for Disease Control and Prevention, the bacteriological culture positivity rate was dramatically higher in Xinjiang, such as 433 per 100,000 compared to the national average level of 119.^[6]^ However, few reports are available about the culture positivity rate and drug resistance of OATB in China.

The present study was conducted to gain insights into the pathogenic bacteria and drug resistance of bone and joint TB in Xinjiang, China, with a view to extend the knowledge base of OATB for developing better TB control strategies.

## 2 Material and methods

### 2.1 Patients and specimens

From January 2017 to December 2018, specimens were collected from a total of 69 patients who were clinically diagnosed with OATB and underwent surgical management; 32 (46.38%) of them were male. The mean age of the patients was 42.4 ± 19.2 (range 6–78). The diagnosis of skeletal TB was established following full clinical, laboratory, and radiological investigation based on the 2015 Infectious Diseases Society of America guideline.^[7]^ There were 57 cases of spinal TB and 12 cases of knee, hip, ankle, elbow, and sacroiliac joint TB. Informed consent was obtained from all subjects included in the study. The protocols and procedures for the protection of human subjects were approved by the Ethics Committee of the First Affiliated Hospital of Xinjiang Medical University; furthermore, all the methods were carried out in accordance with the approved guidelines.

### 2.2 Specimen collection and preservation

During operation, after revealing the lesion of TB, 2 to 20 mL pus was extracted with a 30 mL sterile syringe from abscess of paravertebral, psoas, fossa iliaca, and other parts. If no pus, granulation tissue, necrosis intervertebral disc, and caseous necrosis were extracted. After sealing of specimen bag, they were numbered and registered, then kept in dark place and put at −80°C in a refrigerator for cryopreservation, prepared for inspection. Cryopreservation time was 2 to 209 days, average 79.7 ± 60.7 days.

### 2.3 Instruments and reagents

BACTEC MGIT960 system, oscillator, centrifuge, biosafety cabinet, mycobacterial growth indicator tube (MGIT), oleic acid, albumin, dextrose, and catalase medium, PANTA bacteria inhibitor (an antibiotic mixture of polymyxin B, amphotericin B, nalidixic acid, trimethoprim, and azlocillin), and drug sensitive kit were all provided by Becton, Dickinson and Company, United States. It was reported that the positive coincidence rate of the BACTEC MGIT960 system is about 95% to 100%,^[8]^ and it is a more commonly used clinical detection method. Nosova et al believe that the BACTEC MGIT 960 system is fast effective and reliable when it comes to detecting MDR-TB.^[9]^

### 2.4 Culturing method

For pus, 2 mL of isolate was taken and put into a sterile centrifuge tube; then equal amount of 4% NaOH digestive juice was added. If no pus, tissues like necrotic intervertebral disc, granulation tissue, etc were grinded with mortar and equal amount of 4% NaOH digestive juice was added; then they were oscillated for 30 seconds (if specimens were thick, digestion time was extended appropriately). Placed at room temperature for 10 minutes, supernatant was decanted, 20 mL 0.067 mol/L PBS was added, after being washed 2 times, 0.5 mL 0.067 mol/L PBS was added, mixed and inoculated in MGIT; meanwhile, a direct smear acid-fast staining was done to the inoculum. MGIT was putted into BACTEC 960 system, then the instrument detected automatically. The growth of mycobacteria in the culture tube was judged. If positive result appeared, the instrument prompted automatically. Then positive culture was taken out, and direct smear acid-fast staining was done to confirm whether the mycobacteria.

### 2.5 Species identification

In accordance with the “Laboratory Science Procedure of Diagnostic Bacteriology in Tuberculosis” published by Chinese Antituberculosis Association,^[10]^ the positive strain was inoculated in the thiophene-2-carboxylic acid hydrazine (TCH) and the P-nitro benzoic acid (PNB) medium for species identification. MTB: TCH (+), PNB (–); *M. bovis*: TCH (–), PNB (-); NTM: TCH (+), PNB (+).

### 2.6 Drug susceptibility testing

According to the M24-A mycobacterium trace medium dilution method of American Clinical and Laboratory Standards Institute, the experiment was designed by means of determining the minimum inhibitory drug concentration method; the drug and its concentration was WHO recommended. 0.5 mL of positive culture was inoculated into a MGIT culture tube containing a standard concentration of the drug (including oleic acid, albumin, dextrose, and catalase medium); cultures were diluted 1:5 and inoculated in MGIT culture tubes as growth control; then they were placed in BACTEC MGIT 960 system; the instrument reported the drug sensitivity results automatically according to the *M. bacilli* growth status. Operation of BACTEC MGIT 960 system was in strict accordance with the manufacturer's operating instructions. Drug susceptibility tube and control tube were measured on the same day or the control tube after 1 to 2 days. The one determined as positive by interpretoscope was drug resistant. Two days later, the control tube showed positive; if drug susceptibility tube still not saw bacteria growth, then it was determined as drug sensitivity. A total of 16 drugs were tested: streptomycin (S) 1.0 μg/mL, isoniazid (H) 0.2 μg/mL, rifampicin (R) 1.0 μg/mL, ethambutol (E) 2.0 μg/mL, ofloxacin 2.0 μg/mL, levofloxacin (LFX) 2.0 μg/mL, amikacin 10.0 μg/mL, capreomycin 10.0 μg/mL, propylthiouracil isonicotinic amine 5.0 μg/mL, lectra lung disease 0.2 μg/mL, clarithromycin (CLA) 16.0 μg/mL, ciprofloxacin 1.0 μg/mL, rifabutin 0.5 μg/mL, clofazimine 0.5 μg/mL, linezolid 0.5 μg/mL, and sulfa methoxy-triazine 32 μg/mL.

### 2.7 Quality Control

H37Rv standard sensitive strain was used as quality control.

### 2.8 Statistical treatment

All statistical analyses were performed using Microsoft Office Excel 2007 and Statistical Package for the Social Sciences (SPSS, version 20.0, Chicago, IL). Chi-Squared test was conducted to detect differences between subgroups, *P* < .05 were considered significant.

## 3 Results

### 3.1 Culturing results

Culturing results of 69 isolates are listed in Table 1. The positive cultures underwent through a direct smear acid fast stain. A total of 40 isolates were confirmed as mycobacterium culture-positive, with no sundry bacterial contamination. The total positive rate was 57.97% with an average required incubation time 24.25 ± 11.04 days (13–55 days). Among all positive isolates, 24 (34.78%) were detected as positive within 4 weeks, and a total of 40 (57.97%) isolates were culture-positive when the incubation time was extended to 8 weeks. The longest frozen time of the positive specimens was 202 days. Culture-positive rate of isolates with frozen time > 60 days was 56.41%, compared with the isolates with frozen time < 60 days (60.00%). The culture-positive rates of patients with TB symptoms such as mild fever, night sweats, and obvious weight loss were significantly higher than those without TB symptoms. The culture-positive rate of isolates of which direct smear acid fast stain microscopic examination were positive was higher than that of negative ones. The culture-positive rates of lesions such as sequestrum and granulation were slightly lower than that of fester and pus. The culture-positive rate of spine group was slightly higher than joint group. The culture-positive rates of C-reactive protein and erythrocyte sedimentation rate (ESR) increased groups were both higher than that of normal groups, but there was no statistically significant difference. The culture-positive rate of retreated patients was much lower than that of first-treated patients, due to small sample size, the difference was still not considered as statistically significant.

**Table 1:**
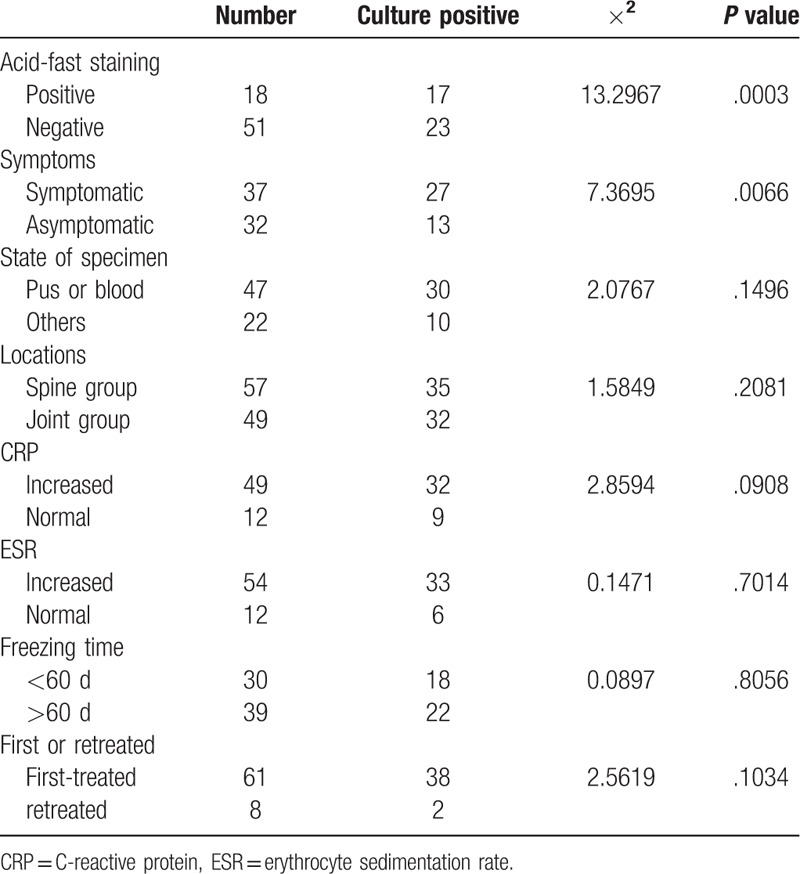
Culturing results of isolations of 69 patients using BACTEC MGIT960 system.

#### 3.1.1 Species identification results

Among the 40 mycobacterial culture positive cases, 24 (60%) cases were MTB, 10 (25%) cases were *M. bovis*, 6 (15%) cases were NTM, and all of the 6 cases were separated from spinal lesions with paraspinal abscess formation, including 3 thoracic TB and 3 lumbar TB, in which 4 cases had TB symptoms (Table 2).

**Table 2:**
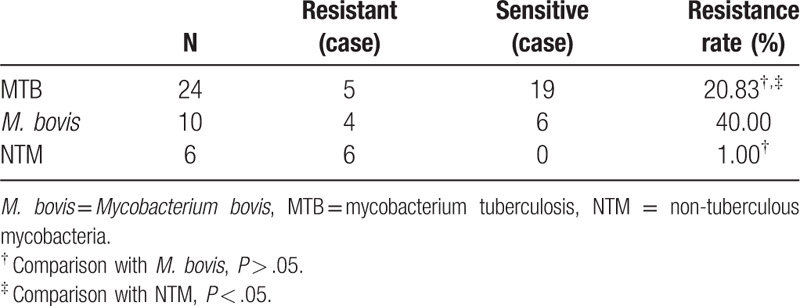
Drug susceptibility test results of 3 kinds of mycobacteria.

#### 3.1.2 Drug resistance results

Forty cases of culture-positive specimens were used for 16 kinds of drug susceptibility tests like H, R, S, E, and so on. In the case of first-line drugs like H, R, S, and E, 25 (62.5%) cases were drug sensitive, 15 (37.5%) cases were drug-resistant, 6 (15%) cases were single drug resistant, 9 cases (22.5%) were multi-drug resistant in which 3 cases (7.5%) were resistant to all first-line drugs as H, R, S, and E. The drug types of 15 drug resistant cases: S 11 cases, H 11 cases, R 4 cases, E 6 cases, ofloxacin 4 cases, LFX 2 cases, amikacin 2 cases, capreomycin 4 cases, propylthiouracil isonicotinic amine 4 cases, lectra lung disease 3 cases, ciprofloxacin 5 cases, rifabutin 2 cases, clofazimine 3 cases, linezolid 1 case, CLA 2 cases, sulfa methoxy-triazine no drug resistant strain. The reporting time of drug sensitivity tests ranged from 5 to 9 days (6.93 ± 1.21 days). The results of drug susceptibility tests of MTB, *M. bovis*, and NTM are listed in Table 2. Paired comparisons were used by calculating the exact probabilities. The drug resistant rate of MTB (20.83%) was significantly lower than that of NTM (100%) (*P* < .05), and the resistant rate of *M. bovis* (40%) was between the two. For the 16 kinds of anti-TB drugs, 6 cases of NTM were resistant up to 8 to 10 drugs and all of them were MDR strains.

## 4 Discussion

In this study, the culture-positive rate was 57.97%, which is considered to be related to the extension of the culturing time; the longest culturing time was 8 weeks. The culture-positive rate of OATB is about 14.5% to 83.87% in China and that of pus is generally 50% to 60%.^[11]^ Since OATB patients generally accepted formal or informal anti-TB drug treatment before surgery, it is needed to extend the culturing time appropriately on the basis of MTB sputum culture. The findings of this study show that the culture positive rate of mycobacteria is related to the acid-fast staining results and the presence of TB symptoms. The material drawn parts, specimen states, C-reactive protein, and ESR have no impact on positive rate.

MTB is found to be the major pathogen of bone and joint TB (60%). However, *M. bovis* and NTM also take up a large proportion. The Fifth National TB Epidemiological Survey of China shows that NTM accounted for 11.1% in isolated mycobacteria strain, *M. bovis* 2.5%.^[12]^ In this study, the proportion of the 2 are both higher than the national average level, and the drug resistance rates are higher, especially the drug resistance rate of NTM increased to 100%, consistent with the report of Zhou et al.^[13]^ The drug susceptibility of NTM depends on mycobacterial type. Slow-growing NTM usually are susceptible to first-line anti-TB drugs.^[14]^

When it comes to its clinical presentation, imaging, and even pathological manifestation, NTM infection is very similar to the MTB infection.^[15]^ Therefore, it is difficult to identify NTM in clinical practice, and misdiagnosed and mistreated cases were reported. Six isolates of NTM infection in this study were all spinal lesions with paraspinal abscess formation, including 3 isolates of thoracic and 3 isolates of lumbar lesions, in which 4 isolates presented TB symptoms; all were diagnosed and treated in accordance with bone TB; clinical outcomes were poor and relapse rates were higher. Among them, a female patient received routine anti-TB drugs and operation, and the disease relapsed 4 times; eventually, it was clinically cured when targeted drugs were used according to the results of drug susceptibility test. Therefore, for the surgical specimens of clinically diagnosed OATB patients, especially for those relapsed cases, mycobacterial culturing, strain identification, and drug resistance analysis should be conducted conventionally, and personalized medicine is recommended based on the results. This also demonstrates that implementing the effective anti-TB drug strategy throughout the entire treatment process is the key to successful treatment, and surgical management cannot replace it.

Some scholars have reported that the interrupted chemotherapy is one of the factors that lead to the relapse of spinal TB after surgery.^[16–18]^ The direct consequence of interrupted chemotherapy is the increase of drug resistance rate. The Fifth National TB Epidemiological Survey of China shows that the total drug resistance of mycobacterium tuberculosis is 36.8% and multi-drug resistant rate is 6.8%.^[12]^ The Drug-Resistant TB Global Monitoring Report 2015 shows that the average drug resistance of new cases is 9.0% (6.5%–11.5%) in 100 countries and regions.^[1]^ In this study, the total drug resistance rate and multi-drug resistance rate are 37.5% and 22.5%, respectively; both are higher than the national average level, most of them resistant to the S and H, consistent with the TB drug resistance monitoring situation in Xinjiang reported by Chen et al.^[19]^

This is a single center, perspective study on mycobacterium culturing and drug resistance of clinically diagnosed OATB patients, which provides a reference for the diagnosis and treatment of OATB in clinical practice. However, there are 2 major limitations in this study that could be addressed in future research. First, the sample size of statistical measurement is insufficient, and the cases may not fully be representative of OATB population. Multi-center, large sample studies would be conducted to make more in-depth analyses. Second, some patients accepted formal or informal treatment previously, and part of patients because of low education level cannot provide accurate medical history and medication use. This may have influence on the drug resistance results. The results of the study highlight the need for future research to use a more representative sample and multidisciplinary collaboration with microbiologists and pharmacologists.

Based on the findings of this study, we suggest that:

1. More cautious steps should be taken when it comes to chemotherapy. Since drug resistance rate in this group of patients was higher than the national average level, chemotherapeutics should be used with the right amounts, depending on the results of drug sensitivity tests. Second-line anti-TB drugs should be used reasonably. CLA is fat-soluble and can easily penetrate into the lipid layer of mycobacterium tuberculosis.^[20]^ Bruhn et al found that the efficacy of chemotherapy regimens containing CLA is better than ordinary chemotherapy regimens in the first 3 months.^[21]^ Ahmad et al revealed that in the treatment of TB, especially in drug resistance cases, quinolones are very effective second-line drugs.^[22]^ In this study, the majority of strains were sensitive to CLA and LFX, and only part of the NTM was resistant to them. Therefore, it is plausible that on the basis of regular first-line anti-TB drugs, CLA and LFX could be added to enhance the efficacy of treatment.
2. More attention should be paid to the NTM infection. According to statistics, a considerable part of NTM infection is misdiagnosed and mistreated clinically as bone and joint TB.^[14]^ The NTM and *M. bovis* infection rates were significantly higher than other regions of the country (Ningxia, Chongqing, Beijing). Since the drug susceptibility of NTM is different from MTB, conventional anti-TB treatment shows poor effects and high relapse rate on NTM.^[14]^ For such patients with NTM, mycobacteria culturing, strain identification, and drug resistance analysis should be carried out to serve as a guide for individual treatment.

## Acknowledgments

We would like to express our deep appreciation to the relevant staff members of orthopedic centers, operating room, clinical laboratory, and specimens repository of the First Affiliated Hospital of Xinjiang Medical University and clinical laboratory of Chest Hospital of Xinjiang Uygur Autonomous Region for their kind help in collecting and processing of the specimens in this study.

## Author contributions

**Conceptualization:** Yeerzati Hajiaheman.

**Formal analysis:** Yi Yang.

**Funding acquisition:** Yeerzati Hajiaheman.

**Investigation:** Yeerzati Hajiaheman, Gele Jin.

**Methodology:** Yi Yang, Gele Jin.

**Project administration:** Gele Jin.

**Software:** Yeerzati Hajiaheman.

**Supervision:** Gele Jin.

**Writing – original draft:** Yeerzati Hajiaheman, Yi Yang.

**Writing – review & editing:** Nuerhanati Shayilanbieke, Gele Jin.
